# Sarcopenia, adiposity and large discordance between cystatin C and creatinine‐based estimated glomerular filtration rate in patients with cancer

**DOI:** 10.1002/jcsm.13469

**Published:** 2024-04-22

**Authors:** Paul E. Hanna, Tianqi Ouyang, Ismail Tahir, Nurit Katz‐Agranov, Qiyu Wang, Lea Mantz, Ian Strohbehn, Daiana Moreno, Destiny Harden, James E. Dinulos, Duru Cosar, Harish Seethapathy, Justin F. Gainor, Sachin J. Shah, Shruti Gupta, David E. Leaf, Florian J. Fintelmann, Meghan E. Sise

**Affiliations:** ^1^ Division of Nephrology, Department of Medicine Medical College of Wisconsin Milwaukee WI USA; ^2^ Division of Nephrology, Department of Medicine Massachusetts General Hospital Boston MA USA; ^3^ Department of Radiology Massachusetts General Hospital Boston MA USA; ^4^ Department of Diagnostic and Interventional Radiology University Medical Center of the Johannes Gutenberg University Mainz Mainz Germany; ^5^ Division of Hematology and Oncology, Department of Medicine Massachusetts General Hospital Boston MA USA; ^6^ Division of General Internal Medicine, Department of Medicine Massachusetts General Hospital Boston MA USA; ^7^ Division of Renal Medicine, Department of Medicine Brigham and Women's Hospital Boston MA USA; ^8^ Adult Survivorship Program Dana‐Farber Cancer Institute Boston MA USA

**Keywords:** cystatin C, eGFR discrepancy, glomerular filtration rate, sarcopenia, serum creatinine

## Abstract

**Background:**

Creatinine‐based estimated glomerular filtration rate (eGFR_CRE_) may overestimate kidney function in patients with sarcopenia. While cystatin C‐based eGFR (eGFR_CYS_) is less affected by muscle mass, it may underestimate kidney function in patients with obesity. We sought to evaluate the relationship between body composition defined by computed tomography (CT) scans and discordance between creatinine, eGFR_CRE_ and eGFR_CYS_ in adult patients with cancer.

**Methods:**

This study is a cross‐sectional study of consecutive adults with cancer with an abdominal CT scan performed within 90 days of simultaneous eGFR_CRE_ and eGFR_CYS_ measurements between May 2010 and January 2022. Muscle and adipose tissue cross‐sectional areas were measured at the level of the third lumbar vertebral body using a validated deep‐learning pipeline. CT‐defined sarcopenia was defined using independent sex‐specific cut‐offs for skeletal muscle index (<39 cm^2^/m^2^ for women and <55 cm^2^/m^2^ for men). High adiposity was defined as the highest sex‐specific quartile of the total (visceral plus subcutaneous) adiposity index in the cohort. The primary outcome was eGFR discordance, defined by eGFR_CYS_ > 30% lower than eGFR_CRE_; the secondary outcome was eGFR_CYS_ > 50% lower than eGFR_CRE_. The odds of eGFR discordance were estimated using multivariable logistic regression modelling. Unadjusted spline regression was used to evaluate the relationship between skeletal muscle index and the difference between eGFR_CYS_ and eGFR_CRE_.

**Results:**

Of the 545 included patients (mean age 63 ± 14 years, 300 [55%] females, 440 [80.7%] non‐Hispanic white), 320 (58.7%) met the criteria for CT‐defined sarcopenia, and 136 (25%) had high adiposity. A total of 259 patients (48%) had >30% eGFR discordance, and 122 (22.4%) had >50% eGFR discordance. After adjustment for potential confounders, CT‐defined sarcopenia and high adiposity were both associated with >30% eGFR discordance (adjusted odds ratio [aOR] 1.90, 95% confidence interval [CI] 1.12–3.24; aOR 2.01, 95% CI 1.15–3.52, respectively) and >50% eGFR discordance (aOR 2.34, 95% CI 1.21–4.51; aOR 2.23, 95% CI 1.19–4.17, respectively). A spline model demonstrated that as skeletal muscle index decreases, the predicted difference between eGFR_CRE_ and eGFR_CYS_ widens considerably.

**Conclusions:**

CT‐defined sarcopenia and high adiposity are both independently associated with large eGFR discordance. Incorporating valuable information from body composition analysis derived from CT scans performed as a part of routine cancer care can impact the interpretation of GFR estimates.

## Introduction

Accurate assessment of the estimated glomerular filtration rate (eGFR) is critical, especially in patients with cancer who commonly receive renally cleared medications with narrow therapeutic indices. Estimating GFR using serum creatinine (SCr) remains the most widely used method in both clinical practice and research.^1^, ^2^ Yet, because creatinine is a byproduct of muscle metabolism, creatinine production is lower in patients with sarcopenia, a common condition affecting patients with cancer.^2^, ^3^ As a result, creatinine levels may be falsely low in patients with sarcopenia and lead to overestimation of GFR. Cystatin C is a low‐molecular‐weight (13 K Dalton) protein produced by all nucleated cells that is filtered by the glomerulus and is not reabsorbed or secreted.^4^ Unlike SCr, cystatin C is not readily affected by age, sex, muscle mass or diet and has been increasingly used as an alternative to SCr to estimate GFR, with some caveats (e.g., cystatin C is elevated in obesity, uncontrolled thyroid disease and acute inflammatory states).S1

Cancer cachexia is a multifactorial syndrome characterized by the loss of skeletal muscle leading to progressive functional impairment, which is strongly associated with increased mortality.^5^ However, recent studies have shown that visibly wasted patients are increasingly rare in clinical practice; a clinical phenotype of ‘sarcopenic obesity’ has emerged to describe patients with a high body mass index (BMI) but with underlying depleted muscle mass.^S2^ Therefore, clinical impressions of cachexia or BMI are inadequate markers for sarcopenia in patients with cancer, and more precise evaluation of body composition using imaging techniques has been increasingly used in clinical practice over the past 15 years.^6^, ^7^ Computed tomography (CT) scans are widely used for body composition assessment^8^, ^9^; the cross‐sectional areas of tissues on single images at the level of the third lumbar vertebra (L3) strongly correlate with whole body adipose tissue, muscle and lean tissue mass.^10^, ^11^ Using different diagnostic thresholds, studies have shown that 40–60% of patients with locally advanced or metastatic cancer had imaging‐defined sarcopenia.^S3^ ^–^ ^S7^ In addition, imaging‐defined sarcopenia has been shown to have a strong association with adverse clinical outcomes in patients with cancer.^12^, ^13^, ^14^

Given that patients with active cancer undergo frequent CT scans for diagnosis and monitoring of disease progression, CT is a convenient tool to evaluate body composition and quantify skeletal muscle and adipose tissue and, thus, may aid in the accurate interpretation of SCr or cystatin C‐based eGFR (eGFR_CRE_ and eGFR_CYS_). Because patients with sarcopenia may have a falsely low SCr and obesity is associated with elevated cystatin C, we hypothesized that CT‐defined sarcopenia and high adiposity will each be associated with a large discordance between eGFR_CRE_ and eGFR_CYS_. Our objective was to evaluate the association between body composition (skeletal muscle index [SMI] and adiposity index) assessed by abdominal CT scan and discordance between eGFR_CRE_ and eGFR_CYS_ in patients with cancer.

## Methods

### Patient population and study design

Using the centralized data warehouse of our health system, the Research Patient Data Registry (RPDR), we designed a retrospective cohort study of consecutive adult patients with a pre‐existing diagnosis of malignancy who had simultaneous measurements of SCr and cystatin C as a part of routine care between May 2010 and January 2022. We defined the baseline as the date of the simultaneous eGFR_CRE_ and eGFR_CYS_ measurements. In patients with multiple simultaneous assessments, the first instance was used. We calculated eGFR_CRE_ using the Chronic Kidney Disease Epidemiology Collaboration (CKD‐EPI) 2021 race‐free equation^2^ and eGFR_CYS_ using the CKD‐EPI 2012 race‐free equation.^15^, ^16^ We included all patients who had an abdominal CT scan performed within 90 days before or after the baseline date. We excluded patients with eGFR_CRE_ < 15 mL/min/1.73 m^2^ and those whose CT scans failed quality control for the determination of SMI (*Table* *S1*).

### Data collection

Comorbidities were defined based on diagnosis codes appearing any time prior to the baseline date. Corticosteroid use was defined by an active prescription within 30 days of the baseline date. The cancer type was determined by the most frequently coded cancer‐related diagnosis prior to the baseline. Cancer stage was determined by chart review and defined as early stage versus locally advanced or advanced (stage 3 or 4) based on oncologists' documentation. Patients who had previously completed antineoplastic treatment or underwent surgery without evidence of disease as documented by their oncologist were considered to be in remission. Baseline chronic kidney disease was defined by the CKD‐EPI 2021 race‐free equation that incorporates both SCr and cystatin C (eGFR_CRE–CYS_ < 60 mL/min/1.73 m^2^).^17^

### Body composition analysis on computed tomography scans

**Figure 1:**
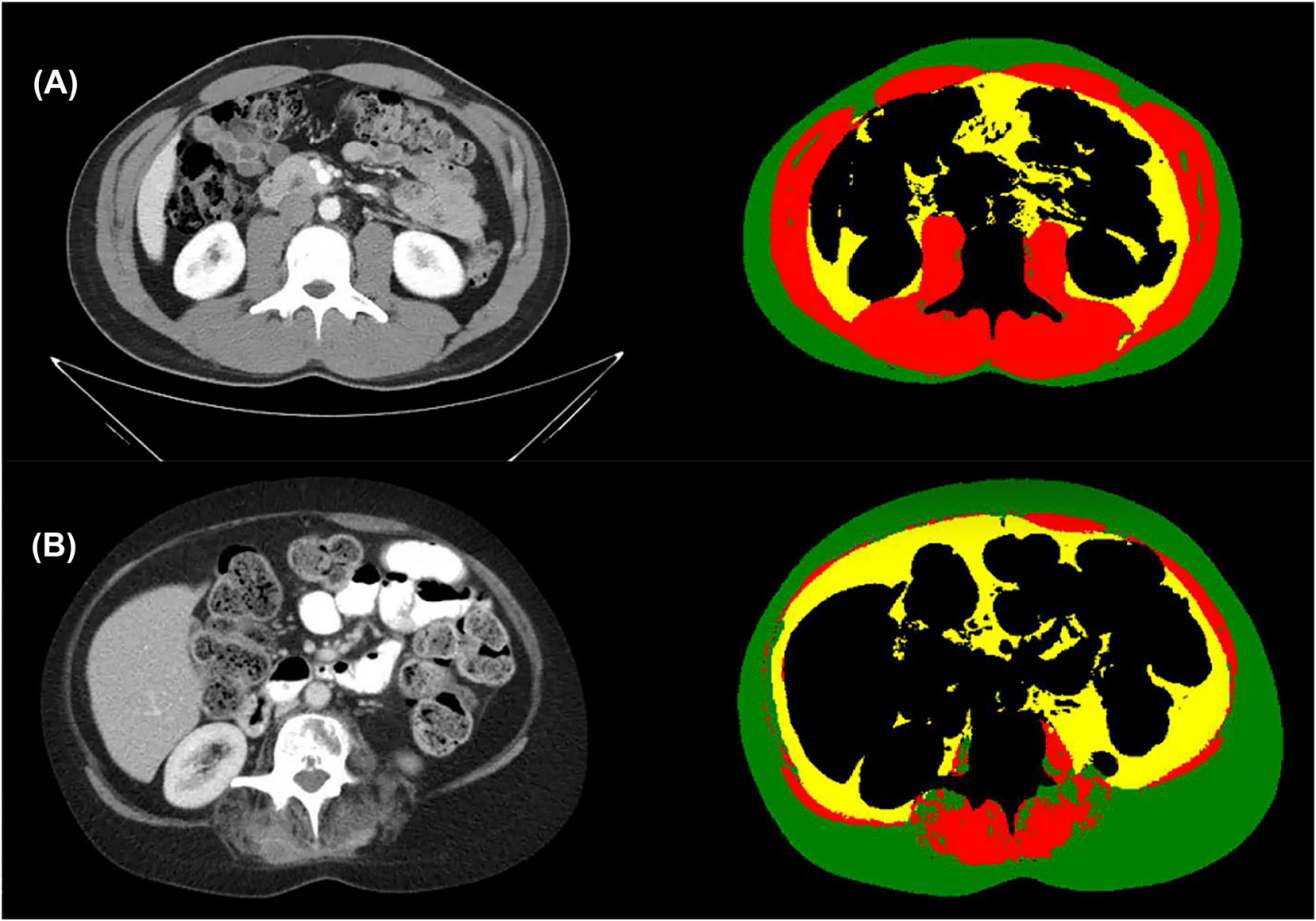
Body composition analysis at the level of the third lumbar vertebral body. Representative axial computed tomography images at the level of the third lumbar vertebral body (left column) and matching segmentation label maps (right column). Patient A was a 26‐ to 30‐year‐old male with a skeletal muscle (red) index (SMI) of 65.9 (4th quartile), a subcutaneous adiposity (green) index (SAI) of 38.6 (2nd quartile) and a visceral adiposity (yellow) index (VAI) of 25.8 (1st quartile). Patient B is a 46‐ to 50‐year‐old female with an SMI (red) of 18.9 (1st quartile), an SAI (green) of 77.3 (2nd quartile) and a VAI (yellow) of 36.92 (3rd quartile).

We used a multistep pipeline, including previously validated machine learning algorithms, to identify the third lumbar vertebral level (L3) on axial images and segment the cross‐sectional area of skeletal muscle and adipose tissue at this level.^6^, ^18^, ^19^, ^20^, ^21^ Each segmentation label map was independently reviewed by a trained analyst with 2 years' experience performing body composition analysis blinded to clinical outcomes, including eGFR_CRE_ and eGFR_CYS_ (IT). We calculated SMI (cm^2^/m^2^) by dividing the skeletal muscle cross‐sectional area by the patient's height squared. Visceral and subcutaneous fat areas were normalized for height (divided by height squared) to calculate the visceral adiposity index (VAI) and the subcutaneous adiposity index (SAI) in cm^2^/m^2^ (*Figure* *1*). The total adiposity index was calculated by adding VAI and SAI.^6^, ^21^ When possible, we selected outpatient CT scans; if no outpatient CT scan was available within 90 days, we included the inpatient CT scan closest to the baseline date.

### Primary exposure

CT‐defined sarcopenia was defined using previously published SMI cut‐offs of <39 cm^2^/m^2^ for women and <55 cm^2^/m^2^ for men.^6^ High adiposity was defined as the highest sex‐specific quartile of total adiposity index (VAI plus SAI), as no cut‐offs for low or high adiposity index exist.

### Primary outcome

The primary outcome was eGFR discordance, defined as eGFR_CYS_ more than 30% lower than the eGFR_CRE_. The reference group consisted of all patients who did not meet the criteria for eGFR discordance. The 30% cut‐off was chosen as it is commonly used in clinical studies to define the accuracy of eGFR compared with measured GFR.^22^, ^23^

### Secondary outcomes

To evaluate a more severe eGFR discordance, we additionally identified a subset of patients with eGFR_CYS_ that was more than 50% lower than eGFR_CRE_. Finally, to assess the absolute eGFR difference (eGFR_DIFF_), we subtracted eGFR_CRE_ from eGFR_CYS_; negative values indicated that the eGFR_CRE_ was higher than the eGFR_CYS_. We evaluated the continuous relationship between eGFR_DIFF_ and SMI. We defined a large eGFR_DIFF_ as having an eGFR_CYS_ more than 15 mL/min/1.73 m^2^ lower than eGFR_CRE_.^24^

### Statistical analysis

We reported baseline characteristics for the entire cohort by SMI quartile using counts and percentages for categorical variables, means with standard deviations (±SD) for normally distributed continuous variables and median and interquartile range (IQR) for skewed variables. Variables with missing values were imputated using the multiple imputation method; five imputed datasets were obtained through chained equations, and parameter estimates from each imputed dataset were pooled using Rubin's rules.^S8^

We examined the unadjusted associations between baseline demographics, comorbidities, medications, laboratory studies and body composition (CT‐defined sarcopenia and high adiposity) with eGFR discordance. We fit a multivariable logistic regression model to determine the association between eGFR discordance (>30% and >50%) and body composition, accounting for potential confounders. For the sensitivity analyses, we fit a multivariable logistic regression model to determine the association between the lowest sex‐specific SMI quartile and >30% eGFR discordance and fit a multivariable logistic regression model to determine the association between CT‐defined sarcopenia and large eGFR_DIFF_ (eGFR_CYS_ more than 15 mL/min/1.73 m^2^ lower than eGFR_CRE_), accounting for potential confounders. Haemoglobin and serum albumin were evaluated in clinically relevant categories. Unadjusted spline regression was performed to evaluate the relationship between SMI and eGFR_DIFF_ (eGFR_CYS_ minus eGFR_CRE_) using the B‐spline basis for cubic polynomial splines with three degrees of freedom.

To address bias, we also performed additional sensitivity analyses excluding patients with inpatient CT scans, liquid tumours (lymphoma, leukaemia or myeloma) and acute kidney injury (AKI) at the time of eGFR_CRE_ and eGFR_CYS_ assessments. AKI was defined as an SCr that was 50% higher than the lowest SCr in the 365 days prior to baseline.

All comparisons were two‐sided, with *P* < 0.05 considered significant. All analyses were performed using R 4.1.1 (R Foundation), SAS 9.4 (SAS Institute) and GraphPad Prism V.9.1.0 (GraphPad Software). The design and reporting of this cohort study followed the Strengthening the Reporting of Observational Studies in Epidemiology (STROBE) guidelines for reporting observational studies.

## Results

### Patient and computed tomography scan characteristics

Of the 1988 patients with cancer originally included in this study, 662 had one or more abdominal CT scans within 90 days of the baseline date. We excluded 46 patients with eGFR_CRE_ < 15 mL/min/1.73 m^2^ and 48 patients whose scans failed to accurately segment skeletal muscle or failed quality control (*Figure* *S1* and *Tables* *S1* and *S2*). The included 545 patients had a median age of 64 years (IQR 55–72), 45% were male and 80.7% were non‐Hispanic white (*Table* *1*). Patients had a wide array of cancer types (*Table* *S3*); the most common cancer types were breast (12.5%), gynaecological (11.4%) and gastrointestinal (10.8%). Most patients (*N* = 338; 62%) had advanced disease. The mean BMI was 27.6 kg/m^2^ (SD ± 7.2). Most CT scans (*N* = 375; 69%) were performed in the outpatient setting, and *N* = 334 (61%) were performed with intravenous contrast.

**Table 1:** Baseline patients' characteristics

Covariates	All patients	1st quartile of SMI	2nd quartile of SMI	3rd quartile of SMI	4th quartile of SMI
	*N* = 545	*N* = 137	*N* = 136	*N* = 136	*N* = 136
Age	64 (55–72)	67 (59–76)	65 (56–73)	64 (54–73)	60 (51–67)
Male sex	245 (45.0%)	62 (45.3%)	61 (44.9%)	61 (44.9%)	61 (44.9%)
Race/ethnicity					
Asian	20 (3.7%)	6 (4.4%)	5 (3.7%)	6 (4.4%)	3 (2.2%)
Black	43 (7.9%)	5 (3.6%)	10 (7.4%)	12 (8.8%)	16 (12%)
Hispanic	19 (3.5%)	1 (0.7%)	4 (2.9%)	11 (8.1%)	3 (2.2%)
White	440 (81%)	116 (85%)	114 (84%)	104 (76%)	106 (78%)
Other	23 (4.2%)	9 (6.6%)	3 (2.2%)	3 (2.2%)	8 (5.9%)
Body mass index					
Normal (18.5–24.9)	190 (34.9%)	57 (41.6%)	54 (39.7%)	58 (42.6%)	21 (15.4%)
Underweight (<18.5)	30 (5.5%)	21 (15.3%)	7 (5.1%)	2 (1.5%)	0 (0%)
Overweight (25–29.9)	164 (30.1%)	40 (29.2%)	42 (30.9%)	44 (32.4%)	38 (27.9%)
Obese (≥30)	161 (29.5%)	19 (13.9%)	33 (24.3%)	32 (23.5%)	77 (56.6%)
Baseline eGFR_CRE–CYS_	46.3 [30.7, 75.3]	42.2 [29.6, 68.0]	44.9 [30.4, 79.5]	47.2 [30.7, 76.8]	49.6 [34.0, 71.5]
Skeletal muscle index (cm^2^/m^2^)	42.2 [36.4, 48.2]	31.8 [28.8, 34.2]	39.5 [38.0, 41.2]	44.9 [42.7, 47.5]	53.9 [49.0, 60.8]
Subcutaneous adipose index (cm^2^/m^2^)	67.1 [42.0, 100.8]	55.4 [34.5, 81.6]	67.2 [42.5, 93.6]	68.3 [39.9, 97.8]	89.3 [55.5, 123.6]
Visceral adipose index (cm^2^/m^2^)	48.5 [22.3, 83.3]	45.6 [20.7, 70.6]	43.5 [20.4, 78.5]	42.1 [19.5, 66.6]	69.4 [32.6, 101.6]
Total adiposity index (cm^2^/m^2^)	125.7 [72.4, 174.0]	106.6 [68.0, 151.9]	122.2 [68.9, 169.2]	114.0 [64.2, 164.5]	156.9 [102.1, 222.4]
Cancer stage					
Advanced/active treatment	338 (62.0%)	78 (56.9%)	85 (62.5%)	88 (64.7%)	87 (64.0%)
Early stage/remission	207 (38.0%)	59 (43.1%)	51 (37.5%)	48 (35.3%)	49 (36.0%)
Comorbidities					
Hypertension	408 (74.9%)	111 (81.0%)	97 (71.3%)	100 (73.5%)	100 (73.5%)
Coronary artery disease	279 (51.2%)	88 (64.2%)	69 (50.7%)	63 (46.3%)	59 (43.4%)
Diabetes mellitus	299 (54.9%)	90 (65.7%)	80 (58.8%)	66 (48.5%)	63 (46.3%)
Cirrhosis	43 (7.9%)	9 (6.6%)	15 (11.0%)	7 (5.1%)	12 (8.8%)
Human immunodeficiency virus	20 (3.7%)	7 (5.1%)	4 (2.9%)	4 (2.9%)	5 (3.7%)
Smoking	235 (43.1%)	69 (50.4%)	57 (41.9%)	58 (42.6%)	51 (37.5%)
Malnutrition	68 (12.5%)	19 (13.9%)	18 (13.2%)	17 (12.5%)	14 (10.3%)
Corticosteroid use	186 (34.1%)	65 (47.4%)	50 (36.8%)	36 (26.5%)	35 (25.7%)
Labs					
Serum albumin (g/dL)					
<3.0	168 (30.8%)	69 (50.4%)	50 (36.8%)	28 (20.6%)	21 (15.4%)
3.0–3.9	183 (33.4%)	46 (33.6%)	44 (32.4%)	49 (36.0%)	44 (32.4%)
≥4.0	194 (35.6%)	22 (16.2%)	42 (30.9%)	59 (43.4%)	71 (52.2%)
SCr (mg/dL)	1.24 [0.85, 1.73]	1.15 [0.66, 1.61]	1.15 [0.76, 1.64]	1.31 [0.89, 1.85]	1.31 [1.02, 1.73]
Serum cystatin C (mg/L)	1.63 [1.12, 2.33]	1.84 [1.32, 2.59]	1.74 [1.05, 2.24]	1.56 [1.06, 2.21]	1.42 [1.07, 2.03]
Haemoglobin (g/dL)					
<10.0	274 (50.3%)	96 (70.1%)	71 (52.2%)	60 (44.1%)	47 (34.6)
10.0–11.9	131 (24.0%)	24 (17.5%)	37 (27.2%)	38 (27.9%)	32 (23.5)
≥12.0	140 (25.7%)	17 (12.4%)	28 (20.6%)	38 (27.9%)	57 (41.9)

*Note*: Counts and percentages or median and interquartile ranges are shown. Missing data: Serum albumin is missing for 4 patients (0.7%), the visceral adipose index is missing for 9 patients (1.7%) and the subcutaneous adipose index is missing for 77 patients (14.1%). Abbreviations: eGFR, estimated glomerular filtration rate; SCr, serum creatinine; SMI, skeletal muscle index.

### Body composition analysis

**Figure 2:**
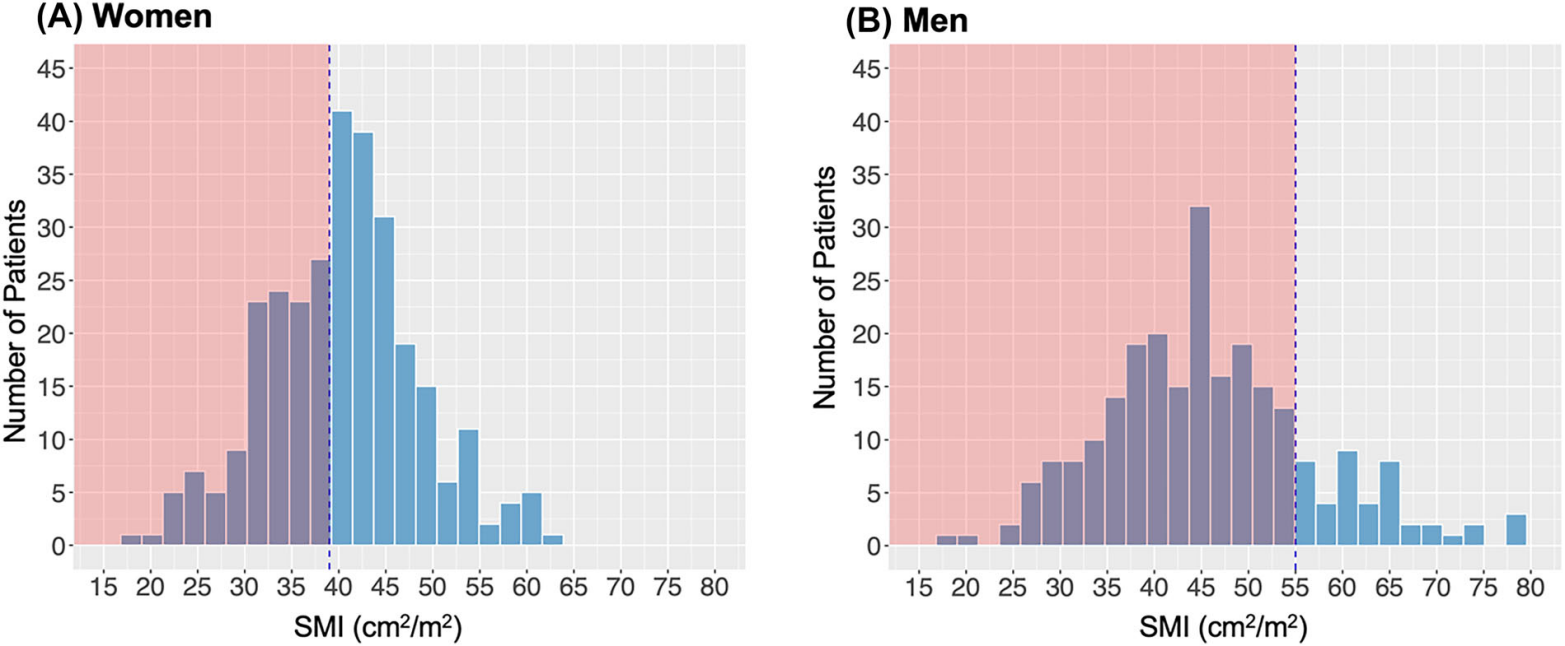
(A, B) Histograms showing the distribution of skeletal muscle index (SMI) by sex. The red shaded area denotes the sex‐specific cut‐offs for sarcopenia as described by Prado et al.^6^

SMI was available for all included patients. A total of 320 (58.7%) patients met predefined sex‐specific SMI thresholds for CT‐defined sarcopenia.^6^ Though mean SMI was higher in men compared with women (45.8 ± 11.7 vs. 40.5 ± 8.6 cm^2^/m^2^; *P* < 0.001), a higher fraction of men met criteria for CT‐defined sarcopenia (81.5% vs. 40%; *P* < 0.001) (*Figure* *2*).

Visceral and subcutaneous fat indices were available for 536 (98%) and 468 (86%) of the cohort, respectively, and both were available for 465 (85%) of the cohort. The mean VAI was higher in men than women (mean 69.5 ± 43.0 vs. 44.7 ± 35.8 cm^2^/m^2^; *P* < 0.001), whereas the mean SAI was higher in women than men (mean 89.0 ± 54.7 vs. 62.1 ± 40.1 cm^2^/m^2^; *P* < 0.001) (*Figure* *S2*).

### Primary and secondary outcomes

**Figure 3:**
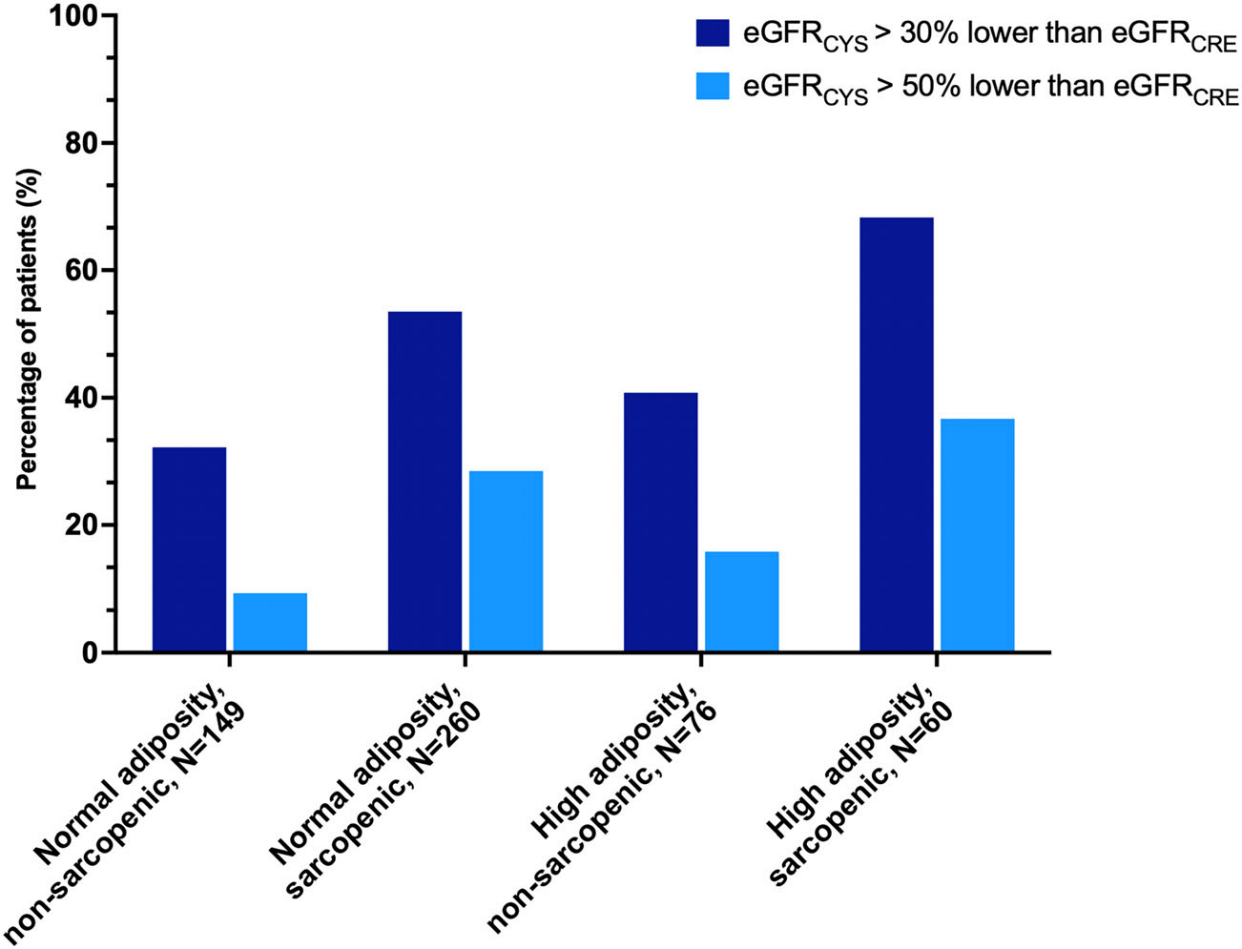
Rate of estimated glomerular filtration rate (eGFR) discrepancy by body composition. The rate of eGFR discrepancy (eGFR_CYS_ > 30% lower than eGFR_CRE_) and severe eGFR discrepancy (eGFR_CYS_ > 50% lower than eGFR_CRE_) by body composition. Sarcopenia is defined by sex‐specific cut‐offs for skeletal muscle index (<39 cm^2^/m^2^ for women and <55 cm^2^/m^2^ for men); high adiposity is defined by the highest sex‐specific quartile.

**Figure 4:**
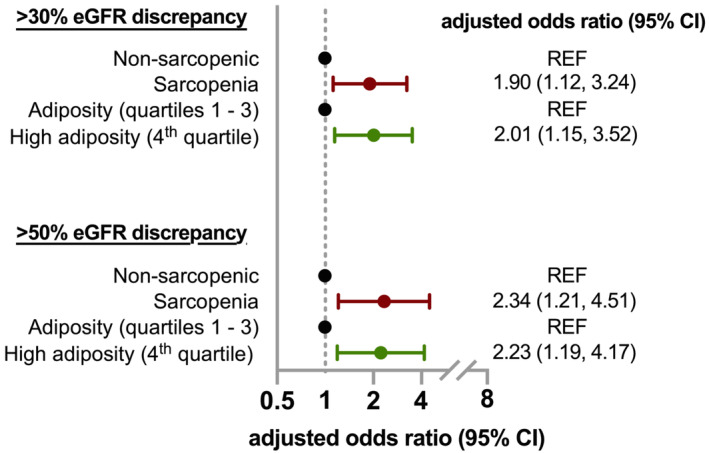
Forest plot illustrating the strength of associations with 30% and 50% estimated glomerular filtration rate (eGFR) discrepancies. The final model for both >30% and >50% eGFR discrepancies is adjusted for the variables shown in *Table* *2*. The unadjusted and multivariable logistic regression model for >50% eGFR discrepancy is shown in *Table* *S4*. CI, confidence interval; REF, reference group.

A total of 259 patients (48%) met the primary outcome of eGFR discordance, defined as eGFR_CYS_ more than 30% lower than the eGFR_CRE_. A scatterplot of eGFR_CRE_ versus eGFR_CYS_ is shown in *Figure* *S3A*, and the distribution of the differences between eGFR_CRE_ and eGFR_CYS_ is shown in *Figure* *S3B*. Among the 320 patients with CT‐defined sarcopenia, 180 (56.3%) met the primary outcome; among the 136 patients with high adiposity, 72 (52.9%) met the primary outcome; and 41 (68.3%) of the 60 patients with both CT‐defined sarcopenia and high adiposity met the primary outcome (*Figure* *3*). In the final multivariable model adjusted for variables shown in *Table* *2*, CT‐defined sarcopenia and high adiposity each remained independently associated with eGFR discordance (adjusted odds ratio [aOR] 1.90, 95% confidence interval [CI] 1.12–3.24, *P* = 0.018; aOR 2.01, 95% CI 1.15–3.52, *P* = 0.014, respectively) (*Figure* *4*). A sensitivity analysis demonstrated an independent association between the lowest sex‐specific SMI quartile and eGFR discordance (aOR 2.34, 95% CI 1.21–4.51, *P* = 0.012; *Table* *S4*).

**Table 2:** Predictors of estimated glomerular filtration rate discrepancy

Covariates	30% eGFR discrepancy (*N* = 259) vs. reference (*N* = 286)					
	Unadjusted			Multivariable		
	OR	95% CI	*P*‐value	Adjusted OR	95% CI	*P*‐value
Age (per 10 years)	1.15	1.02, 1.30	0.024	0.92	0.77, 1.10	0.344
Male sex	1.33	0.95, 1.87	0.1	0.69	0.41, 1.15	0.154
Baseline eGFR_CRE–CYS_	0.98	0.97, 0.98	<0.001	0.98	0.97, 0.99	<0.001
Sarcopenia	2.38	1.67, 3.38	<0.001	1.90	1.12, 3.24	0.018
Highest adiposity quartile	1.32	0.89, 1.96	0.160	2.01	1.15, 3.52	0.014
Liquid vs. solid tumour	2.83	1.78, 4.48	<0.001	1.51	0.80, 2.84	0.201
Acute kidney injury	1.93	1.37, 2.73	<0.001	0.56	0.34, 0.92	0.022
Inpatient vs. outpatient CT scan	4.60	3.09, 6.84	<0.001	1.81	1.09, 3.00	0.021
Smoking	1.54	1.09, 2.17	0.014	1.11	0.70, 1.76	0.649
Comorbidities						
Hypertension	2.57	1.70, 3.88	<0.001	1.00	0.54, 1.87	0.988
Coronary artery disease	2.65	1.87, 3.75	<0.001	1.08	0.67, 1.74	0.760
Diabetes mellitus	4.03	2.81, 5.79	<0.001	1.42	0.86, 2.33	0.170
Cirrhosis	4.65	2.18, 9.92	<0.001	3.04	1.22, 7.59	0.017
HIV	1.69	0.68, 4.21	0.26			
Malnutrition	1.68	1.00, 2.82	0.048	1.02	0.53, 1.96	0.957
Thyroid disease	1.52	1.04, 2.24	0.032	0.97	0.59, 1.61	0.920
Medication use^a^						
Corticosteroids	3.57	2.45, 5.19	<0.001	1.71	1.06, 2.76	0.029
Labs						
Albumin (g/dL)						
≥4.0	REF	—		—	—	
3.0–3.99	5.58	3.47, 8.99	<0.001	3.28	1.79, 6.02	<0.001
<3.0	15.6	9.28, 26.1	<0.001	9.00	4.46, 18.2	<0.001
Haemoglobin (g/dL)						
≥12.0	REF	—				
10.0–11.99	1.70	1.00, 2.89	0.052	0.73	0.36, 1.47	0.375
<10.0	6.31	3.97, 10.0	<0.001	1.22	0.61, 2.45	0.566

*Note*: eGFR discrepancy is defined by eGFR_CYS_ > 30% lower than eGFR_CRE_. Baseline eGFR_CRE–CYS_ was defined using the Chronic Kidney Disease Epidemiology Collaboration 2021 race‐free combined cystatin C and creatinine equation. Abbreviations: CI, confidence interval; CT, computed tomography; eGFR, estimated glomerular filtration rate; HIV, human immunodeficiency virus; OR, odds ratio; REF, reference.

^a^ Corticosteroid use was defined within 30 days of baseline.

**Figure 5:**
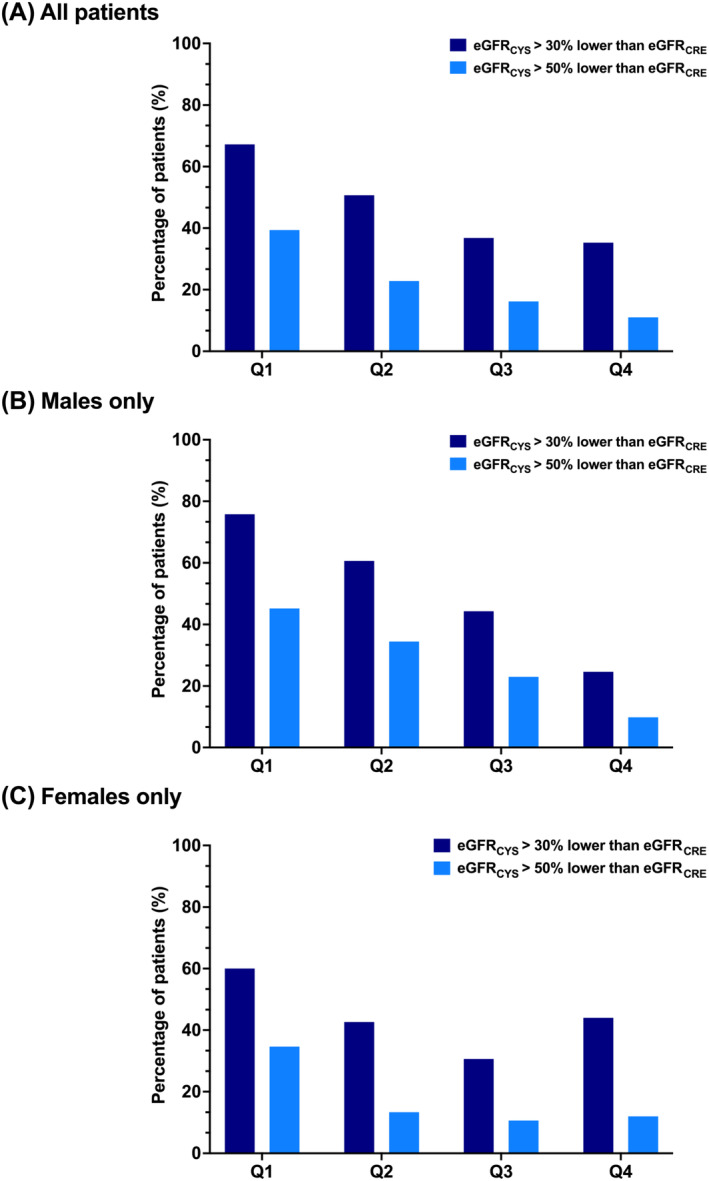
(A–C) Rates of 30% and 50% estimated glomerular filtration rate (eGFR) discrepancies by skeletal muscle index (SMI) quartile. Rates of >30% and >50% eGFR discrepancies by quartiles of SMI. Lower SMI values (Q1) indicate a greater degree of sarcopenia. Q1, 1st quartile; Q2, 2nd quartile; Q3, 3rd quartile; Q4, 4th quartile.

A total of 122 patients (22.4%) had >50% eGFR discordance (eGFR_CYS_ > 50% lower than eGFR_CRE_). CT‐defined sarcopenia and high adiposity were also strongly associated with having an eGFR_CYS_ more than 50% lower than eGFR_CRE_ (aOR 2.34, 95% CI 1.21–4.51, *P* = 0.012; aOR 2.23, 95% CI 1.19–4.17, *P* = 0.013, respectively) (*Figure* *4* and *Table* *S5*). The prevalence of 30% and 50% eGFR discordance by sex‐specific SMI quartile is shown in *Figure* *5* *A* and stratified by sex in *Figure* *5* *B,C*.

**Figure 6:**
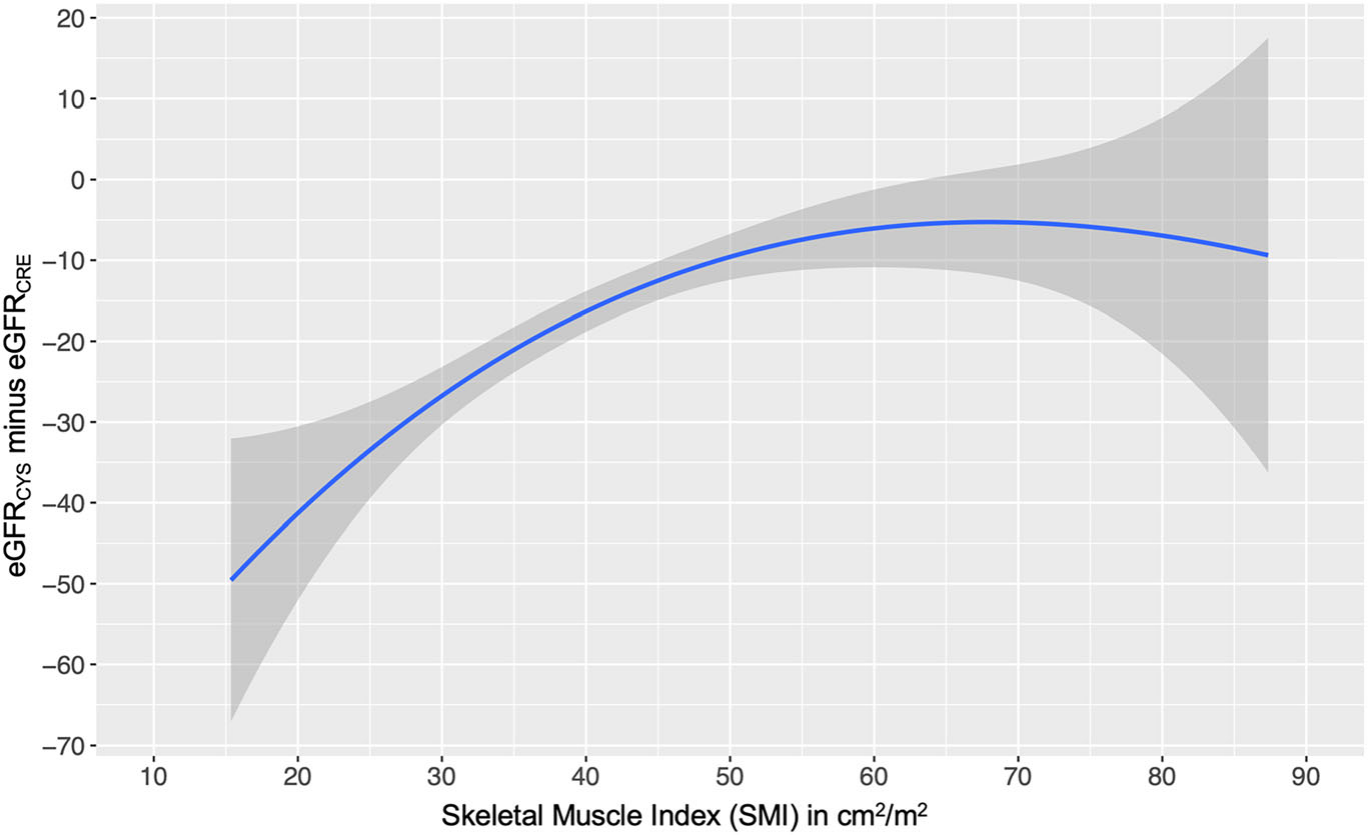
Spline model predicting the relationship between skeletal muscle index (SMI) and estimated glomerular filtration rate (eGFR) difference (eGFR_CYS_ minus eGFR_CRE_). Unadjusted spline regression was performed to evaluate the relationship between SMI and eGFR_DIFF_ (eGFR_CYS_ minus eGFR_CRE_) using the B‐spline basis for cubic polynomial splines with three degrees of freedom using the 10th to 90th SMI percentile for the cohort. Lower SMI values indicate a greater degree of sarcopenia.

We evaluated the relationship between CT‐defined sarcopenia and the eGFR differences (eGFR_DIFF_) defined by subtracting eGFR_CRE_ from eGFR_CYS_ (negative values mean that eGFR_CYS_ is lower than eGFR_CRE_). The mean eGFR_DIFF_ was −20.9 ± 25.1 mL/min/1.73 m^2^ in patients with CT‐defined sarcopenia compared with −8.6 ± 22.4 mL/min/1.73 m^2^ in patients without CT‐defined sarcopenia (*P* < 0.001), and 247 (45%) of patients met the definition of large eGFR_DIFF_ (eGFR_CYS_ more than 15 mL/min/1.73 m^2^ lower than eGFR_CRE_). A sensitivity analysis also demonstrated an independent association between CT‐defined sarcopenia and a large eGFR difference (aOR 1.97, 95% CI 1.13–3.43, *P* = 0.017; *Table* *S6*). A spline model showing the continuous relationship between SMI and eGFR_DIFF_ demonstrated that as SMI decreased, the predicted difference between eGFR_CRE_ and eGFR_CYS_ widened considerably (*Figure* *6*).

### Sensitivity analyses

Exclusion of patients with AKI, inpatient CT scans or liquid tumours did not meaningfully alter the relationship between SMI quartiles and eGFR discordance (*Figure* *S4A–C*, respectively).

## Discussion

In a cohort of patients with cancer, we found that CT‐defined sarcopenia and high adiposity, defined by CT scan, are independent risk factors for eGFR discordance. Furthermore, the lower a patient's SMI, the wider the discordance between eGFR_CRE_ and eGFR_CYS_. Accurate assessment of eGFR is important but challenging in patients with cancer due to the high prevalence of sarcopenia and sarcopenic obesity. Patients with cancer are commonly exposed to antineoplastic therapies or supportive medications (antibiotics, anticonvulsants and anticoagulants) that require dose adjustments based on kidney function; therefore, a large discordance between eGFR_CRE_ and eGFR_CYS_ poses significant challenges in clinical management and may be associated with adverse medication events and lower survival.^25^

Sarcopenia is associated with decreased survival in patients with cancer and has been associated with increased treatment‐related toxicity in patients receiving antineoplastic therapies.^26^, ^27^, ^28^, ^29^ We hypothesize that medication overdose due to inaccurate estimation of GFR when relying on eGFR_CRE_ in patients with sarcopenia may cause dose‐related toxicity in renally excreted chemotherapies^26^ and may thereby contribute to the decreased survival in these patients. Body composition analysis using CT scans is a more sensitive and specific way of assessing the quantity and distribution of skeletal muscle and adipose tissue than BMI.^6^, ^10^, ^11^

Prior studies have suggested that obesity may increase cystatin C levels independent of kidney function, which leads to an underestimation of GFR using the cystatin C equation. However, this observation is largely based on obesity defined by BMI rather than a detailed analysis of body composition.^30^ Prior studies utilized clinical and anthropomorphic measurements (BMI, muscle strength, skin fold thickness, etc.) as an assessment of sarcopenia and obesity, which may not as accurately reflect the body composition of muscle and adipose tissue.^S9^ ^–^ ^S11^ As cystatin C is secreted by nucleated cells and constitutively expressed in almost all organs,^31^ higher body cell mass, which correlates with a higher BMI, could lead to a higher cystatin C level independent of kidney function. A study in children showed that adjustment with body cell mass, estimated by bioimpedance, increased the accuracy of cystatin C‐based GFR estimation.^32^ Other studies have suggested that increased cystatin C mRNA expression in adipose tissue may be contributing to higher cystatin C levels in obese individuals.^33^ To our knowledge, this is the first study that demonstrated an association between CT‐defined total adiposity index and a higher odds of discordance between eGFR_CRE_ and eGFR_CYS_.

Additionally, we found that patients with liquid tumours have significantly higher rates of eGFR discordance than patients with solid tumours. This possibly reflects a higher cancer cell burden in liquid tumours that may lead to higher cystatin C levels,^34^ suggesting that there is a complex relationship between serum biomarkers and individual disease states, and future studies are needed to best individualize GFR estimation in patients with cancer.

Other recent studies demonstrate an association between large differences in eGFR_CRE_ and eGFR_CYS_ and adverse clinical outcomes in non‐cancer populations, including frailty, falls, hospitalizations and death.^35^, ^36^ Of note, the rate of large eGFR discordance in our cohort was significantly higher than prior studies conducted in populations without cancer (*Figure* *S3B*), suggesting that the high prevalence of sarcopenia in patients with cancer is an important risk factor for discordance between eGFR_CRE_ and eGFR_CYS_.^35^, ^36^ A recent study of 327 patients in China with resectable gastric cancer showed an association between a lower serum creatine/cystatin C ratio and the presence of imaging‐defined sarcopenia.^37^ Our cohort included patients with various tumour types and stages and found an independent relationship between both CT‐defined sarcopenia and high adiposity with eGFR discordance. A study of 22 316 US veterans who had a simultaneous SCr and cystatin C showed that patients whose creatinine to cystatin C ratio was ≤0.75 had a higher mortality risk (3.03 [2.81–3.27]) compared with those with a creatinine to cystatin C ratio of 1.00 to <1.25; this affirms that the relationship between creatinine and cystatin C (or eGFR_CRE_ and eGFR_CYS_) may be an important indicator of health status independent of kidney function.^38^

Our study has several limitations. First, cystatin C was ordered as part of clinical care, which likely selected a population in whom clinicians questioned the accuracy of creatine‐based eGFR; therefore, the rate of eGFR discordance in our study is likely to be higher than in the general cancer population. Second, our study included a significant number of patients who were hospitalized or had unstable kidney function at the time of their CT scan, suggesting that a significant fraction of patients were experiencing acute illness. However, sensitivity analyses excluding patients with inpatient CT scans and AKI did not meaningfully change the relationship between CT‐defined sarcopenia and eGFR discordance. Third, we used a single measurement of SCr and cystatin C, which may not reflect a steady state at the time of measurement. Although these may limit the generalizability of our findings, these variables were adjusted for in the multivariable model. Fourth, a subset of patients either did not have a CT scan or were excluded during quality assurance. Of note, automated segmentation was not possible in patients with soft tissue oedema, which particularly affects the subcutaneous adipose tissue metric. Fifth, we evaluated adiposity quartiles as previously used by others^39^; however, this may affect the generalizability of our definition of ‘high adiposity’ across other populations. Sixth, CT‐defined sarcopenia does not quantify muscle quality or strength, important aspects of the clinical definition of sarcopenia that more accurately predict adverse outcomes.^40^ Finally, our study lacks the gold standard (measured GFR) given the retrospective design; prospective studies are needed to validate the accuracy of GFR estimating equations in consecutive patients with sarcopenia and high adiposity against measured GFR.

In conclusion, we demonstrate that CT‐defined sarcopenia is associated with a higher risk of eGFR discordance; as SMI decreases, the discordance between eGFR_CRE_ and eGFR_CYS_ widens considerably. High adiposity is also associated with a higher risk of large eGFR discordances. Deep learning models facilitate fully automated body composition analysis on abdominal CT scans done as a part of routine cancer care, allowing for the potential for widespread clinical use in the future. Patients with altered body composition may require measured GFR to determine an accurate GFR for drug dosing.^6^, ^10^, ^11^, ^21^ Future studies that account for body composition are needed to improve and personalize the approach to GFR estimation in patients with cancer who rely on accurate eGFR to guide chemotherapy dosing.

## Conflict of interest statement

SG reports research support from BTG International and GE Healthcare. She is a member of GlaxoSmithKline's Global Anemia Council, a consultant for Secretome and the founder of the American Society of Onconephrology. MES reports research funding from Gilead, Merck, EMD Serono, Otsuka, Novartis and Angion. She has served as a scientific advisory board member for Mallinckrodt, Travere, Vera and Novartis and is a data monitoring committee member for Alpine Immunosciences. DEL reports research support from BioPorto, BTG International and Metro International Biotech LLC. FJF has received research funding from the William M. Wood Foundation and Pfizer and has a related patent WO2019051358A1. JFG has served as a compensated consultant or received honoraria from Bristol‐Myers Squibb, Genentech/Roche, Takeda, Loxo/Lilly, Blueprint Medicine, Gilead, Moderna, AstraZeneca, Curie Therapeutics, Mirati, Merus Pharmaceuticals, Nuvalent, Pfizer, Novartis, Merck, iTeos, Karyopharm, Silverback Therapeutics and Glyde Bio; has received research support from Novartis, Genentech/Roche and Takeda; has received institutional research support from Bristol‐Myers Squibb, Tesaro, Moderna, Blueprint, Jounce, Array Biopharma, Merck, Adaptimmune, Novartis and Alexo; and has an immediate family member who is an employee with equity at Ironwood Pharmaceuticals. All remaining authors have no conflicts of interest.

## Supporting information


**Table S1.** Reasons for body composition analysis failure
**Table S2.** Comparison of baseline characteristics of patients with excluded vs. included scans
**Table S3.** Cancer types by skeletal muscle index quartile (SMI), in alphabetical order
**Table S4.** Sensitivity analysis using lowest sex specific SMI quartile as a predictor of eGFR_CYS_ more than 30% lower than eGFR_CRE_
**Table S5.** Predictors of eGFR_CYS_ more than 50% lower than eGFR_CRE_
**Table S6.** Predictors of eGFR_CYS_ more than 15 mL/min/1.73m^2^ lower than eGFR_CRE_
**Figure S1.** Patient flow
**Figure S2.** Sex‐stratified histograms of subcutaneous and visceral adiposity indices
**Figure S3.** Scatter plot of creatinine‐based and cystatin C‐based eGFR, and distribution of eGFR difference
**Figure S4.** Sensitivity analyses

## Data Availability

The data that support the findings of this study are available from the Research Patient Data Registry at Mass General Brigham, but restrictions apply as they were used under licence for the current study and so are not publicly available. De‐identified data are available from Dr. Meghan Sise upon reasonable request via email and after the execution of a data use agreement with Mass General Brigham.
