# Supplementary material for: Sarcopenia, adiposity and large discordance between cystatin C and creatinine‐based estimated glomerular filtration rate in patients with cancer
Source: J Cachexia Sarcopenia Muscle. 2024 Apr 22;15(3):1187–98. doi: 10.1002/jcsm.13469 (PMC11154767; doi:10.1002/jcsm.13469)
Supplement: Supplementary file 1 — Table S1. Reasons for body composition analysis failure Table S2. Comparison of baseline characteristics of patients with excluded vs. included scans Table S3. Cancer types by skeletal muscle index quartile (SMI), in alphabetical order Table S4. Sensitivity analysis using lowest sex specific SMI quartile as a predictor of eGFRCYS more than 30% lower than eGFRCRE Table S5. Predictors of eGFRCYS more than 50% lower than eGFRCRE Table S6. Predictors of eGFRCYS more than 15 mL/min/1.73m2 lower than eGFRCRE Figure S1. Patient flow Figure S2. Sex‐stratified histograms of subcutaneous and visceral adiposity indices Figure S3. Scatter plot of creatinine‐based and cystatin C‐based eGFR, and distribution of eGFR difference Figure S4. Sensitivity analyses [file JCSM-15-1187-s001.docx]

**Supplemental Appendix**

**Table of content**

**Supplemental Tables**

Supplemental Table 1. ………………………………………………………………………………………2

Supplemental Table 2. ………………………………………………………………………………………3

Supplemental Table 3. ………………………………………………………………………………………4

Supplemental Table 4. ………………………………………………………………………………………5

Supplemental Table 5. ………………………………………………………………………………………6

Supplemental Table 6. ...…………………………………………………………………………………….7

**Supplemental Figures**

Supplemental Figure 1. ………………………………………………………………………………………6

Supplemental Figure 2. ………………………………………………………………………………………7

Supplemental Figure 3. ………………………………………………………………………………………8

Supplemental Figure 4. ………………………………………………………………………………………9

**Supplemental References** ………………………………………………………………………………..10

**Supplemental Table 1. Reasons for body composition analysis failure**

| Reason | N = 48 |
| --- | --- |
| 1. Artifacts due to low dose technique, motion, or image reconstruction | 23 |
| 1. Soft tissue edema | 9 |
| 1. Small field of view | 6 |
| 1. Artifacts from surgical hardware, drains, or abdominal wall hernia | 6 |
| 1. Artifacts from patient skin touching CT gantry | 4 |

**Supplemental Table 1.** There were 48 patients whose scans were excluded from body composition analysis for reasons listed in the above table.

**Supplemental Table 2. Comparison of baseline characteristics of patients with excluded vs. included scans**

| Covariates | Excluded scans | Included scans |
| --- | --- | --- |
|  | N = 48 | N = 545 |
| Age, years | 72 [62, 79] | 63 [55, 72] |
| Male Sex | 22 (45.8%) | 245 (45.0%) |
| White Race | 32 (66.7%) | 440 (80.7%) |
| Race/ethnicity |  |  |
| Asian | 3 (6.2%) | 20 (3.7%) |
| Black | 6 (12%) | 43 (7.9%) |
| Hispanic | 5 (10%) | 19 (3.5%) |
| White | 32 (67%) | 440 (81%) |
| Other | 2 (4.2%) | 23 (4.2%) |
| Normal (18.5 - 24.9) | 13 (27.1%) | 190 (34.9%) |
| Underweight (<18.5) | 2 (4.2%) | 30 (5.5%) |
| Overweight (25 - 29.9) | 27 (56.2%) | 164 (30.1%) |
| Obese (≥30) | 6 (12.5%) | 161 (29.5%) |
| Baseline eGFR_CRE-CYS_ | 43.9 [23.6, 69.8] | 46.3 [30.7, 75.3] |
| Advanced/Active treatment | 25 (52.1%) | 338(62%) |
| Early-stage/Remission | 23 (47.9%) | 207 (38.0%) |
| Comorbidities |  |  |
| Hypertension | 43 (89.6%) | 408 (74.9%) |
| Coronary Artery Disease | 36 (75.0%) | 279 (51.2%) |
| Diabetes Mellitus | 34 (70.8%) | 299 (54.9%) |
| Cirrhosis | 4 (8.3%) | 43 (7.9%) |
| Human Immunodeficiency Virus | 1 (2.1%) | 20 (3.7%) |
| Smoking | 19 (39.6%) | 235 (43.1%) |
| Malnutrition | 10 (20.8%) | 68 (12.5%) |
| Labs |  |  |
| Serum Albumin (g/dL) |  |  |
| <3.0 | 35 (72.9%) | 168 (30.8%) |
| 3.0-3.9 | 7 (14.6%) | 183 (33.6%) |
| $\boldsymbol{\geq}$4.0 | 6 (12.5%) | 194 (35.6%) |
| Serum Creatinine (mg/dL) | 1.06 [0.72, 1.68] | 1.24 [0.85, 1.73] |
| Serum Cystatin C (mg/L) | 1.96 [1.12, 3.12] | 1.63 [1.12, 2.33] |
| Hemoglobin (g/dL) |  |  |
| $\mathbf{<}$10.0 | 34 (70.8%) | 282 (50.3%) |
| 10.0 - 11.9 | 9 (18.8%) | 123 (22.6%) |
| $\boldsymbol{\geq}$12.0 | 5 (10.4%) | 140 (25.7%) |

**Supplemental Table 2**. The baseline characteristics of the 48 patients whose scans were excluded from body composition analysis are compared to the included cohort. The reason for scan exclusion is shown in **Supplemental Table 1**.

**Supplemental Table 3. Cancer types by skeletal muscle index quartile (SMI), in alphabetical order**

| Cancer Type | Overall | 1^st^ Quartile of SMI | 2^nd^ Quartile of SMI | 3^rd^ Quartile of SMI | 4^th^ Quartile of SMI |
| --- | --- | --- | --- | --- | --- |
|  | N= 545 | N= 137 | N= 136 | N= 136 | N= 136 |
| Brain | 8 (1.5%) | 1 (0.7%) | 1 (0.7%) | 3 (2.2%) | 3 (2.2%) |
| Breast | 68 (12%) | 12 (8.8%) | 23 (17%) | 15 (11%) | 18 (13%) |
| Gastrointestinal | 59 (11%) | 15 (11%) | 15 (11%) | 15 (11%) | 14 (10%) |
| Genitourinary Cancer | 55 (10.1%) | 10 (7.3%) | 16 (11.8%) | 12 (8.8%) | 17 (12.5%) |
| Gynecological | 62 (11%) | 12 (8.8%) | 12 (8.8%) | 20 (15%) | 18 (13%) |
| Head & Neck cancer | 12 (2.2%) | 4 (2.9%) | 3 (2.2%) | 1 (0.7%) | 4 (2.9%) |
| Leukemia | 54 (9.9%) | 20 (15%) | 14 (10%) | 17 (12%) | 3 (2.2%) |
| Lung cancer | 51 (9.4%) | 17 (12%) | 13 (9.6%) | 11 (8.1%) | 10 (7.4%) |
| Lymphoma | 46 (8.4%) | 13 (9.5%) | 15 (11%) | 5 (3.7%) | 13 (9.6%) |
| Melanoma | 4 (0.7%) | 1 (0.7%) | 3 (2.2%) | 0 (0%) | 0 (0%) |
| Non-melanomatous skin cancer | 41 (7.5%) | 12 (8.8%) | 6 (4.4%) | 8 (5.9%) | 15 (11%) |
| Renal cell carcinoma | 39 (7.2%) | 9 (6.6%) | 5 (3.7%) | 17 (12%) | 8 (5.9%) |
| Sarcoma | 1 (0.2%) | 1 (0.7%) | 0 (0%) | 0 (0%) | 0 (0%) |
| Thyroid cancer | 8 (1.5%) | 2 (1.5%) | 4 (2.9%) | 0 (0%) | 2 (1.5%) |
| Other | 37 (6.8%) | 8 (5.8%) | 6 (4.4%) | 12 (8.8%) | 11 (8.1%) |

**Supplemental Table 3.** Cancer types by skeletal muscle index quartile (SMI). *Primary cancer type was determined by the type most frequently coded in the electronic health record.

**Supplemental Table 4. Sensitivity analysis using lowest sex specific SMI quartile as a predictor of eGFR_CYS_ more than 30% lower than eGFR_CRE_**

| Covariates | eGFR_CYS_ > 30% lower than eGFR_CRE_  (N=259 vs. N=286 [reference]) | | | | | |
| --- | --- | --- | --- | --- | --- | --- |
|  | **Univariable** | | | **Multivariable** | | |
|  | **OR** | **95% CI** | **p-value** | **Adj OR** | **95% CI** | **p-value** |
| Age (per 10 years) | 1.15 | 1.02, 1.30 | 0.024 | 0.92 | 0.77, 1.10 | 0.356 |
| Male Sex | 1.33 | 0.95, 1.87 | 0.1 | 0.89 | 0.56, 1.41 | 0.624 |
| Baseline eGFR | 0.98 | 0.97, 0.98 | <0.001 | 0.98 | 0.97, 0.99 | <0.001 |
| Lowest SMI quartile | **2.95** | **1.96, 4.44** | **<0.001** | **2.15** | **1.24, 3.71** | **0.006** |
| Highest adiposity quartile | 1.32 | 0.89, 1.96 | 0.16 | 1.99 | 1.15, 3.44 | 0.015 |
| Liquid vs. solid tumor | 2.83 | 1.78, 4.48 | <0.001 | 1.55 | 0.83, 2.92 | 0.171 |
| Acute kidney injury | 1.93 | 1.37, 2.73 | <0.001 | 0.59 | 0.36, 0.97 | 0.037 |
| Inpatient vs. Outpatient CT scan | 4.6 | 3.09, 6.84 | <0.001 | 1.8 | 1.08, 2.99 | 0.024 |
| Smoking | 1.54 | 1.09, 2.17 | 0.014 | 1.09 | 0.69, 1.73 | 0.712 |
| Comorbidities | | | | | | |
| Hypertension | 2.57 | 1.70, 3.88 | <0.001 | 0.97 | 0.52, 1.81 | 0.92 |
| Coronary Artery Disease | 2.65 | 1.87, 3.75 | <0.001 | 1.05 | 0.65, 1.70 | 0.841 |
| Diabetes Mellitus | 4.03 | 2.81, 5.79 | <0.001 | 1.48 | 0.90, 2.44 | 0.122 |
| Cirrhosis | 4.65 | 2.18, 9.92 | <0.001 | 3.14 | 1.25, 7.86 | 0.015 |
| HIV | 1.69 | 0.68, 4.21 | 0.26 |  |  |  |
| Malnutrition | 1.68 | 1.00, 2.82 | 0.048 | 1 | 0.52, 1.93 | 0.992 |
| Thyroid disease | 1.52 | 1.04, 2.24 | 0.032 | 0.96 | 0.58, 1.59 | 0.866 |
| Medication Use | | | | | | |
| Corticosteroids | 3.57 | 2.45, 5.19 | <0.001 | 1.7 | 1.05, 2.76 | 0.03 |
| Labs | | | | | | |
| Albumin (g/dL) |  |  |  |  |  |  |
| $\boldsymbol{\geq}$4.0 | — | — |  | — | — |  |
| 3.0-3.9 | 5.58 | 3.47, 8.99 | <0.001 | 3.37 | 1.84, 6.18 | <0.001 |
| <3.0 | 15.6 | 9.28, 26.1 | <0.001 | 8.96 | 4.43, 18.1 | <0.001 |
| Hemoglobin (g/dL) |  |  |  |  |  |  |
| $\boldsymbol{\geq}$12.0 | — | — |  | — | — |  |
| 10.0 - 11.9 | 1.7 | 1.00, 2.89 | 0.052 | 0.74 | 0.37, 1.49 | 0.401 |
| $\boldsymbol{<}$10.0 | 6.31 | 3.97, 10.0 | <0.001 | 1.19 | 0.59, 2.37 | 0.629 |

**Supplemental Table 4.** Baseline eGFR was defined using the race-free CKD Epi 2021 combined cystatin C and creatinine equation. Abbreviations: CI = confidence interval, REF = reference, HIV = human immunodeficiency virus.

**Supplemental Table 5. Predictors of eGFR_CYS_ more than 50% lower than eGFR_CRE_**

| Covariates | eGFR_CYS_ >50% lower than eGFR_CRE_  (N=122 vs. N=423 [reference]) | | | | | |
| --- | --- | --- | --- | --- | --- | --- |
|  | **Univariable** | | | **Multivariable** | | |
|  | **OR** | **95% CI** | **p-value** | **Adj OR** | **95% CI** | **p-value** |
| Age (per 10 years) | 1.04 | 0.90,1.20 | 0.619 | 0.82 | 0.66, 1.01 | 0.057 |
| Male Sex | 1.83 | 1.22,2.75 | 0.004 | 1.12 | 0.63, 2.00 | 0.696 |
| Baseline eGFR | 0.98 | 0.97,0.98 | <0.001 | 0.98 | 0.97, 0.99 | <0.001 |
| Sarcopenia | 3.28 | 2.04,5.27 | <0.001 | 2.34 | 1.21, 4.51 | 0.012 |
| Highest adiposity quartile | 1.28 | 0.80,2.02 | 0.300 | 2.23 | 1.19, 4.17 | 0.013 |
| Liquid vs. solid tumor | 3.50 | 2.19,5.57 | <0.001 | 1.69 | 0.89, 3.22 | 0.110 |
| Acute kidney injury | 2.30 | 1.53,3.47 | <0.001 | 0.71 | 0.41, 1.24 | 0.226 |
| Inpatient vs. Outpatient CT scan | 4.56 | 2.98,6.97 | <0.001 | 2.04 | 1.19, 3.51 | 0.010 |
| Smoking | 1.56 | 1.04,2.34 | 0.0320 | 1.34 | 0.79, 2.28 | 0.284 |
| Comorbidities | | | | | | |
| Hypertension | 2.10 | 1.23,3.58 | 0.007 | 0.72 | 0.32, 1.64 | 0.438 |
| Coronary Artery Disease | 3.31 | 2.12,5.16 | <0.001 | 1.80 | 1.00, 3.24 | 0.049 |
| Diabetes Mellitus | 5.47 | 3.29,9.09 | <0.001 | 2.17 | 1.14, 4.11 | 0.018 |
| Cirrhosis | 4.21 | 2.22,7.97 | <0.001 | 3.19 | 1.43, 7.11 | 0.005 |
| HIV | 2.40 | 0.96,6.03 | 0.062 |  |  |  |
| Malnutrition | 1.53 | 0.87,2.70 | 0.140 |  |  |  |
| Thyroid disease | 1.30 | 0.83,2.03 | 0.245 |  |  |  |
| Medication Use | | | | | | |
| Corticosteroids | 2.98 | 1.97,4.51 | <0.001 | 1.04 | 0.61, 1.77 | 0.897 |
| Labs | | | | | | |
| Albumin (g/dL) |  |  |  |  |  |  |
| $\boldsymbol{\geq}$4.0 | REF | — |  | — | — |  |
| 3.0-3.9 | 8.17 | 3.36, 19.9 | <0.001 | 3.92 | 1.34, 11.4 | 0.013 |
| <3.0 | 27.1 | 11.4, 64.7 | <0.001 | 11.9 | 4.03, 35.0 | <0.001 |
| Hemoglobin (g/dL) |  |  |  |  |  |  |
| $\boldsymbol{\geq}$12.0 | REF | — |  | — | — |  |
| 10.0 - 11.9 | 2.13 | 0.87, 5.22 | 0.097 | 0.72 | 0.24,2.18 | 0.565 |
| $\boldsymbol{<}$10.0 | 9.33 | 4.38, 19.9 | <0.001 | 1.47 | 0.54, 4.03 | 0.455 |

**Supplemental Table 5.** Baseline eGFR was defined using the race-free CKD Epi 2021 combined cystatin C and creatinine equation. Abbreviations: CI = confidence interval, REF = reference, ACEi/ARB = angiotensin converting enzyme inhibitor or angiotensin II receptor blockade, HIV = human immunodeficiency virus.

**Supplemental Table 6. Predictors of eGFR_CYS_ more than 15mL/min/1.73m^2^ lower than eGFR_CRE_**

| Covariates | eGFR_CYS_ >15mL/min/1.73m^2^ lower than eGFR_CRE_  (N=247 vs. N=298 [reference]) | | | | | |
| --- | --- | --- | --- | --- | --- | --- |
|  | **Univariable** | | | **Multivariable** | | |
|  | **OR** | **95% CI** | **p-value** | **Adj OR** | **95% CI** | **p-value** |
| Age (per 10 years) | 0.95 | 0.84, 1.07 | 0.366 | 0.91 | 0.78, 1.07 | 0.264 |
| Male Sex | 1.06 | 0.76, 1.49 | 0.734 | 0.64 | 0.41, 1.01 | 0.057 |
| Baseline eGFR | 1.00 | 0.99, 1.00 | 0.76 | 1.01 | 1.00, 1.02 | 0.029 |
| Sarcopenia | 2.18 | 1.53, 3.10 | <0.001 | 1.78 | 1.12, 2.82 | 0.015 |
| Highest adiposity quartile | 1.09 | 0.74, 1.62 | 0.661 |  |  |  |
| Liquid vs. solid tumor | 2.99 | 1.89, 4.73 | <0.001 | 1.97 | 1.13, 3.43 | 0.017 |
| Acute kidney injury | 1.26 | 0.89, 1.78 | 0.187 |  |  |  |
| Inpatient vs. Outpatient CT scan | 2.56 | 1.76, 3.71 | <0.001 | 1.41 | 0.89, 2.23 | 0.137 |
| Smoking | 1.25 | 0.89, 1.76 | 0.194 |  |  |  |
| Comorbidities | | | | | | |
| Hypertension | 1.77 | 1.18, 2.64 | 0.006 | 1.67 | 0.97, 2.87 | 0.063 |
| Coronary Artery Disease | 1.91 | 1.35, 2.69 | <0.001 | 1.27 | 0.82, 1.96 | 0.281 |
| Diabetes Mellitus | 2.31 | 1.63, 3.28 | <0.001 | 1.24 | 0.79, 1.96 | 0.349 |
| Cirrhosis | 4.44 | 2.14, 9.22 | <0.001 | 2.84 | 1.26, 6.38 | 0.012 |
| HIV | 1.5 | 0.61, 3.68 | 0.379 |  |  |  |
| Malnutrition | 0.95 | 0.57, 1.58 | 0.831 |  |  |  |
| Thyroid disease | 1.22 | 0.83, 1.79 | 0.311 |  |  |  |
| Medication Use | | | | | | |
| Corticosteroids | 2.6 | 1.81, 3.74 | <0.001 | 1.43 | 0.93, 2.20 | 0.1 |
| Labs | | | | | | |
| Albumin (g/dL) |  |  |  |  |  |  |
| $\boldsymbol{\geq}$4.0 | — | — |  | — | — |  |
| 3.0-3.9 | 3.26 | 2.08, 5.11 | <0.001 | 2.63 | 1.49, 4.64 | <0.001 |
| <3.0 | 8.52 | 5.29, 13.7 | <0.001 | 6.16 | 3.26, 11.7 | <0.001 |
| Hemoglobin (g/dL) |  |  |  |  |  |  |
| $\boldsymbol{\geq}$12.0 | — | — |  | — | — |  |
| 10.0 - 11.9 | 1.22 | 0.73, 2.03 | 0.441 | 0.56 | 0.31, 1.04 | 0.067 |
| $\boldsymbol{<}$10.0 | 3.07 | 1.99, 4.74 | <0.001 | 0.63 | 0.33, 1.18 | 0.145 |

**Supplemental Table 6.** Baseline eGFR was defined using the race-free CKD Epi 2021 combined cystatin C and creatinine equation. Abbreviations: CI = confidence interval, REF = reference, HIV = human immunodeficiency virus.

**Supplemental Figure 1. Patient flow**

**
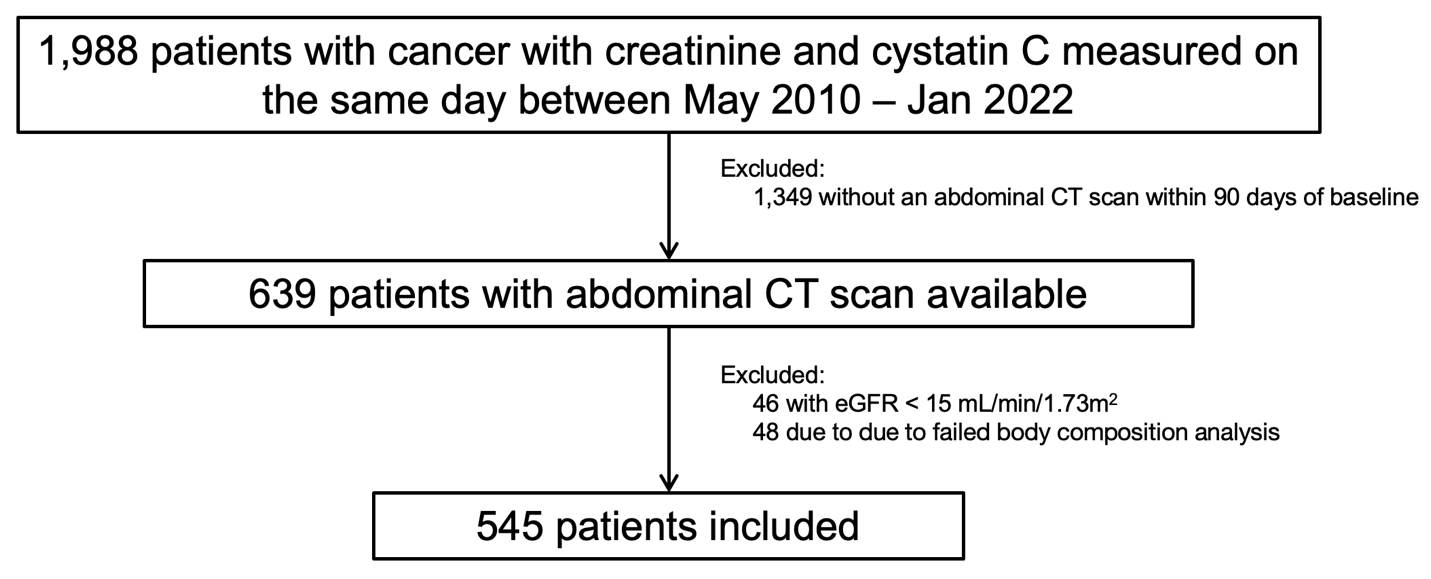
**

**Supplemental Figure 1.** Abbreviations: CT = computed tomography, eGFR = estimated glomerular filtration rate.

**Supplemental Figure 2. Sex-stratified histograms of subcutaneous and visceral adiposity indices**

**
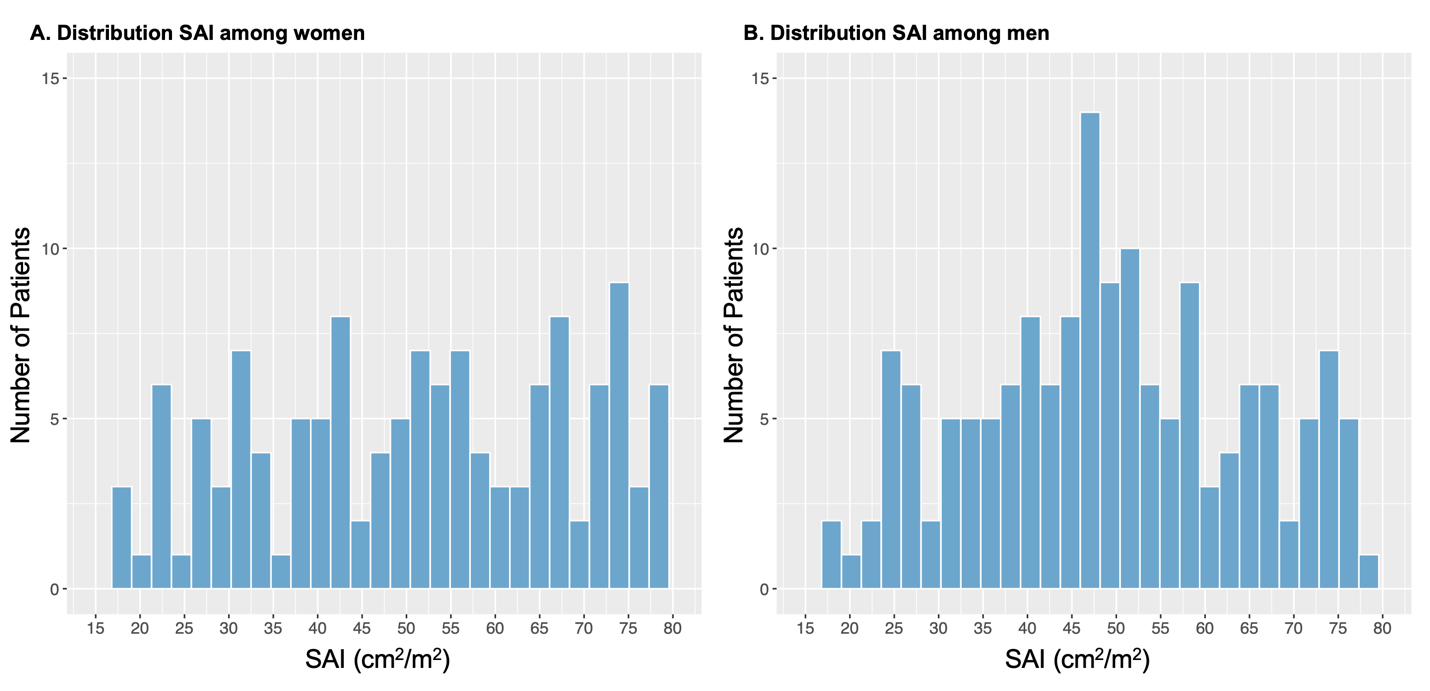
**

**
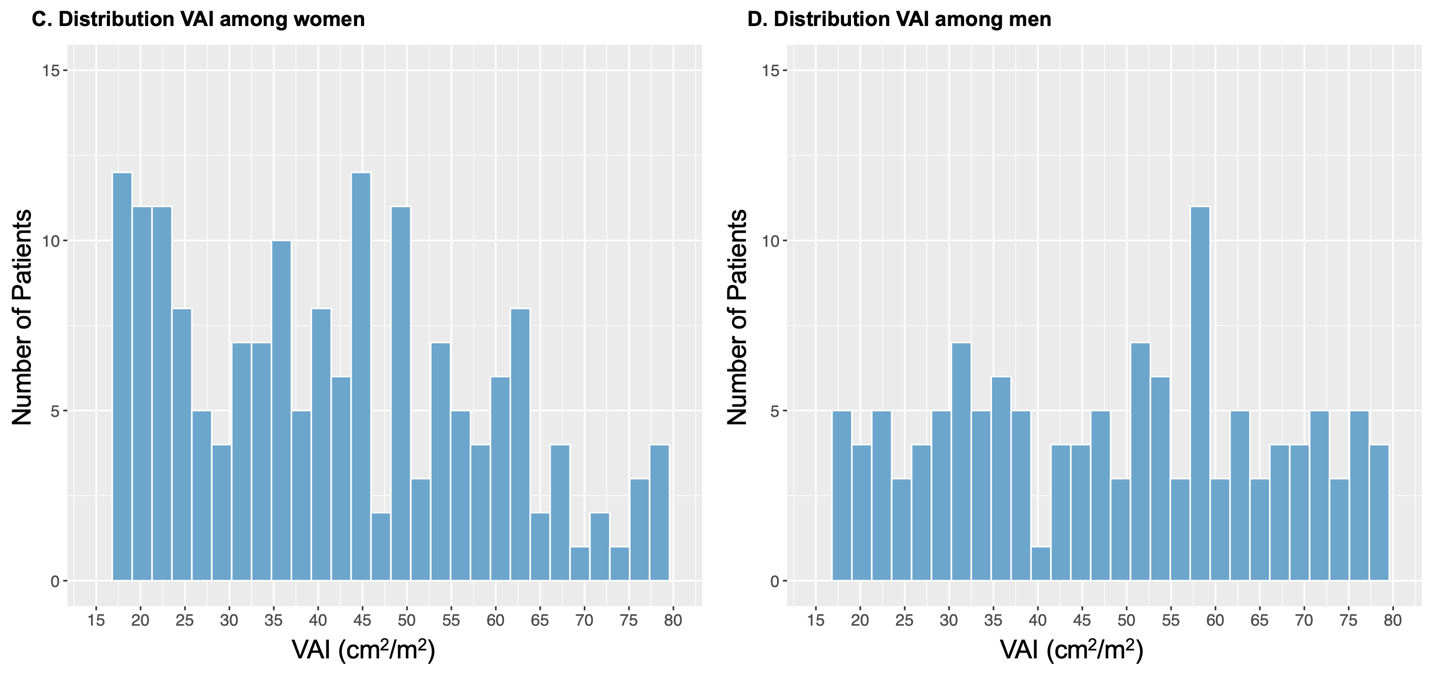
**

**Supplemental Figure 2.** Subcutaneous and visceral adiposity stratified by sex. Abbreviations; SAI = subcutaneous adiposity index, VAI = visceral adiposity index.

**Supplemental Figure 3. Scatter plot of creatinine-based and cystatin C-based eGFR, and distribution of eGFR difference**

**
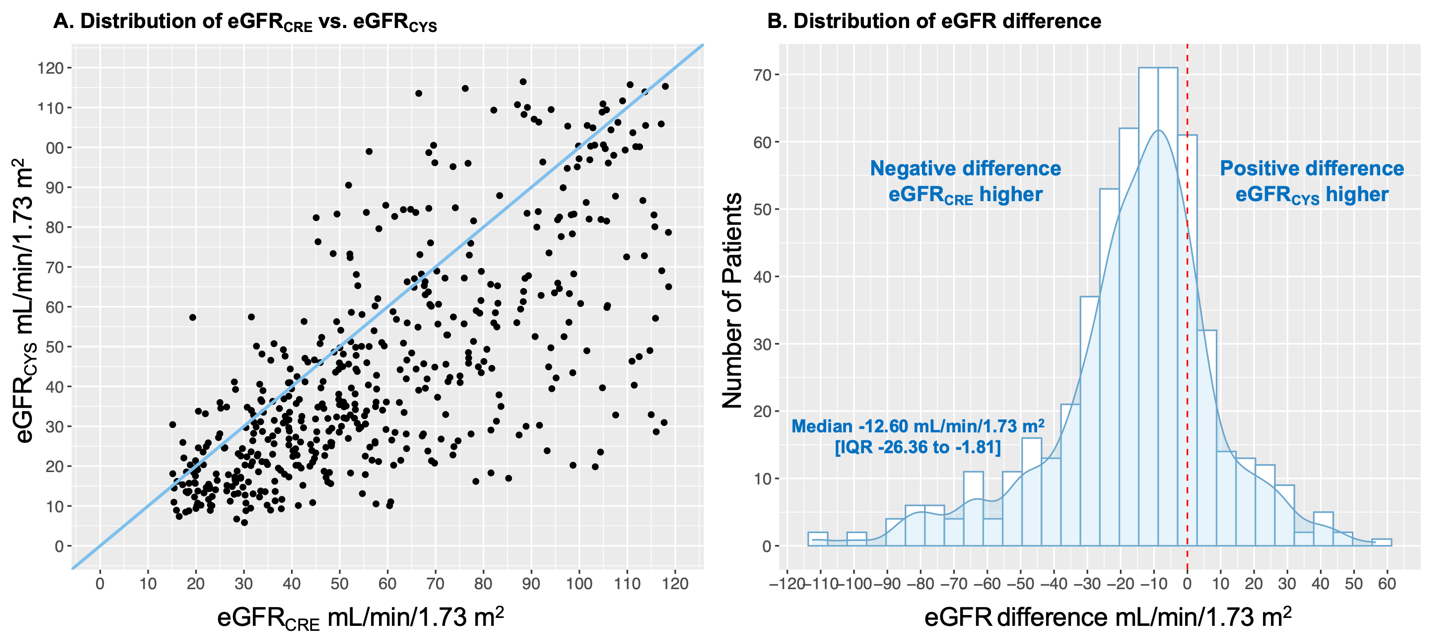
**

**Supplemental Figure 3.** Figure S3A. Scatterplot showing distribution of eGFR_CRE_ and eGFR_CYS_ among study patients; the blue line is the line of equality. Figure S3B. A histogram and superimposed density curve showing the distribution of eGFR difference defined by eGFR_CYS_ minus eGFR_CRE_. The red dotted line signifies equivalence between eGFR_CRE_ and eGFR_CYS_. Abbreviations: eGFR = estimated glomerular filtration rate, IQR = interquartile range

**Supplemental Figure 4. Sensitivity analyses**

**
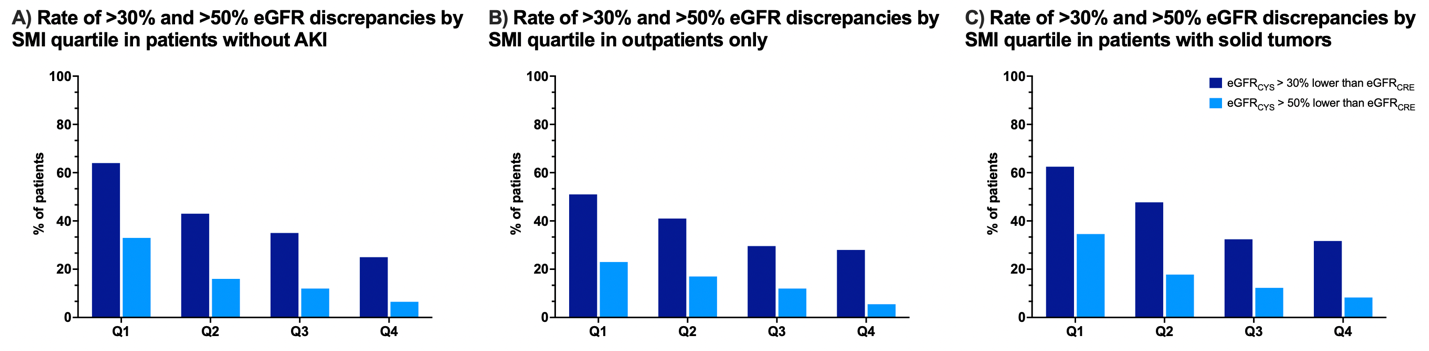
**

**Supplemental Figure 4.** S4A. Sensitivity analysis in patients without acute kidney injury at time of creatinine/cystatin C check, N=319. S4B. patients with outpatient scans, N = 375 and S4C. patients with solid tumors only N = 445 (5C). Abbreviations: eGFR = estimated glomerular filtration rate, SMI = skeletal muscle index, AKI = acute kidney injury, Q1 = first quartile, Q2 = second quartile, Q3 = third quartile, Q4 = 4^th^ quartile.

**Supplemental References:**

1. Knight EL, Verhave JC, Spiegelman D, Hillege HL, de Zeeuw D, Curhan GC, de Jong PE. Factors influencing serum cystatin C levels other than renal function and the impact on renal function measurement. Kidney Int. 2004;65(4):1416-21. doi: 10.1111/j.1523-1755.2004.00517.x. PubMed PMID: 15086483.
2. Prado CM, Lieffers JR, McCargar LJ, Reiman T, Sawyer MB, Martin L, Baracos VE. Prevalence and clinical implications of sarcopenic obesity in patients with solid tumours of the respiratory and gastrointestinal tracts: a population-based study. Lancet Oncol. 2008;9(7):629-35. Epub 20080606. doi: 10.1016/S1470-2045(08)70153-0. PubMed PMID: 18539529.
3. Dalal S, Hui D, Bidaut L, Lem K, Del Fabbro E, Crane C, Reyes-Gibby CC, Bedi D, Bruera E. Relationships among body mass index, longitudinal body composition alterations, and survival in patients with locally advanced pancreatic cancer receiving chemoradiation: a pilot study. J Pain Symptom Manage. 2012;44(2):181-91. Epub 2012/06/15. doi: 10.1016/j.jpainsymman.2011.09.010. PubMed PMID: 22695045; PMCID: PMC3990439.
4. Malietzis G, Currie AC, Athanasiou T, Johns N, Anyamene N, Glynne-Jones R, Kennedy RH, Fearon KC, Jenkins JT. Influence of body composition profile on outcomes following colorectal cancer surgery. Br J Surg. 2016;103(5):572-80. Epub 2016/03/21. doi: 10.1002/bjs.10075. PubMed PMID: 26994716.
5. Rier HN, Jager A, Sleijfer S, van Rosmalen J, Kock MCJM, Levin M-D. Low muscle attenuation is a prognostic factor for survival in metastatic breast cancer patients treated with first line palliative chemotherapy. The Breast. 2017;31:9-15. doi: <https://doi.org/10.1016/j.breast.2016.10.014>.
6. Cushen SJ, Power DG, Murphy KP, McDermott R, Griffin BT, Lim M, Daly L, MacEneaney P, K OS, Prado CM, Ryan AM. Impact of body composition parameters on clinical outcomes in patients with metastatic castrate-resistant prostate cancer treated with docetaxel. Clin Nutr ESPEN. 2016;13:e39-e45. Epub 2017/05/23. doi: 10.1016/j.clnesp.2016.04.001. PubMed PMID: 28531567.
7. Sandini M, Patiňo M, Ferrone CR, Alvarez-Pérez CA, Honselmann KC, Paiella S, Catania M, Riva L, Tedesco G, Casolino R, Auriemma A, Salandini MC, Carrara G, Cristel G, Damascelli A, Ippolito D, D’Onofrio M, Lillemoe KD, Bassi C, Braga M, Gianotti L, Sahani D, Fernández-del Castillo C. Association Between Changes in Body Composition and Neoadjuvant Treatment for Pancreatic Cancer. JAMA Surgery. 2018;153(9):809-15. doi: 10.1001/jamasurg.2018.0979.
8. Rubin D. Multiple Imputation for Nonresponse in Surveys. New York, NY: JohnWiley & Sons. Inc; 1987.
9. Yoshida S, Suda G, Ohara M, Fu Q, Yang Z, Hosoda S, Kimura M, Akinori K, Tokuchi Y, Yamada R, Kitagataya T, Suzuki K, Kawagishi N, Nakai M, Sho T, Natsuizaka M, Morikawa K, Ogawa K, Maehara O, Ohnishi S, Sakamoto N. Frequency and Characteristics of Overestimated Renal Function in Japanese Patients with Chronic Liver Disease and Its Relation to Sarcopenia. Nutrients. 2021;13(7). Epub 2021/08/11. doi: 10.3390/nu13072415. PubMed PMID: 34371925; PMCID: PMC8308887.
10. Williams GR, Dunne RF, Giri S, Shachar SS, Caan BJ. Sarcopenia in the Older Adult With Cancer. J Clin Oncol. 2021;39(19):2068-78. Epub 2021/05/28. doi: 10.1200/jco.21.00102. PubMed PMID: 34043430; PMCID: PMC8260902
11. Baracos VE, Arribas L. Sarcopenic obesity: hidden muscle wasting and its impact for survival and complications of cancer therapy. Ann Oncol. 2018;29(suppl_2):ii1-ii9. Epub 2018/03/06. doi: 10.1093/annonc/mdx810. PubMed PMID: 29506228.
